# Determining the energetic and informational components of speech-on-speech masking

**DOI:** 10.1121/1.4954748

**Published:** 2016-07-11

**Authors:** Gerald Kidd, Christine R. Mason, Jayaganesh Swaminathan, Elin Roverud, Kameron K. Clayton, Virginia Best

**Affiliations:** Department of Speech, Language and Hearing Sciences and Hearing Research Center, Boston University, 635 Commonwealth Avenue, Boston, Massachusetts 02215, USA

## Abstract

Identification of target speech was studied under masked conditions consisting of two or four independent speech maskers. In the reference conditions, the maskers were colocated with the target, the masker talkers were the same sex as the target, and the masker speech was intelligible. The comparison conditions, intended to provide release from masking, included different-sex target and masker talkers, time-reversal of the masker speech, and spatial separation of the maskers from the target. Significant release from masking was found for all comparison conditions. To determine whether these reductions in masking could be attributed to differences in energetic masking, ideal time-frequency segregation (ITFS) processing was applied so that the time-frequency units where the masker energy dominated the target energy were removed. The remaining target-dominated “glimpses” were reassembled as the stimulus. Speech reception thresholds measured using these resynthesized ITFS-processed stimuli were the same for the reference and comparison conditions supporting the conclusion that the amount of energetic masking across conditions was the same. These results indicated that the large release from masking found under all comparison conditions was due primarily to a reduction in informational masking. Furthermore, the large individual differences observed generally were correlated across the three masking release conditions.

## I. INTRODUCTION

Determining the factors that govern the success of a listener attempting to understand the speech of one specific talker in the presence of competing talkers has long been of great interest to auditory and speech scientists (e.g., Miller, 1947; Cherry, 1953; Pollack and Pickett, 1958; Schubert and Schultz, 1962; reviews in Webster, 1983; Yost, 1997; Ebata, 2003; Bronkhorst, 2000, 2015; Mattys *et al.*, 2012; Carlile, 2014). Unfortunately, the literature addressing this topic varies widely in many important respects and contradictory findings and incongruous conclusions are commonplace. As an example, there are large disparities in the literature concerning the presence and degree of release from masking that occurs when the masker speech is simply time reversed. Historically, some influential studies reported negligible improvements in performance when masker speech was temporally reversed compared to when it was presented naturally (i.e., “time forward”, e.g., Schubert and Schultz, 1962; Dirks and Bower, 1969; Festen and Plomp, 1990; Hygge *et al.*, 1992; Summers and Mollis, 2004). The minor effect of time reversal—which renders the speech from a given masker talker unintelligible without changing many of the long-term average characteristics of the source—may be considered similar to reports of only a small beneficial effect of masker speech presented to the listener in a foreign language compared to a primary language (e.g., Freyman *et al.*, 2001; Cooke *et al.*, 2008; Calandruccio *et al.*, 2010). The apparent irrelevance of the intelligibility of speech maskers led Miller (1947) to observe “A language was chosen [as the masker] which the listeners did not know, but the masking was neither greater nor less than was obtained with an English babble [for English speaking listeners]. Once again, it is necessary to conclude that the crucial factor is the masking spectrum. The particular way in which the spectrum is produced is of secondary importance” (p. 120).

Miller's work was influential, and it was many years before this basic premise was challenged. However, more recently, a number of studies have found quite substantial reductions in masking due to masker time reversal. Freyman *et al.* (2001), Marrone *et al.* (2008), Iyer *et al.* (2010), Best *et al.* (2012), Gallun *et al.* (2013), and Swaminathan *et al.* (2015) have all reported large improvements in masked speech reception thresholds (often greater than 10 dB) when intelligible speech maskers were time reversed. Thus, it clearly is the case that there are large discrepancies in the literature with respect to the effect of masker time reversal.

However, the lack of clarity about the role of masker time reversal in speech-on-speech (SOS) masking is not unique when considering stimulus variables that provide a masking release. To varying degrees, similar observations may be made regarding the disparities found in the literature concerning the consequences of such factors as spatial separation of sources, maskers presented in a listener's primary vs secondary vs unknown language, and sex differences between target and masker talkers, among others (e.g., Freyman *et al.*, 2001, 2004; Brungart *et al.*, 2001; Van Engen and Bradlow, 2007; Iyer *et al.*, 2010; Calandruccio *et al.*, 2013; Gallun *et al.*, 2013; reviews in Schneider *et al.*, 2007; Mattys *et al.*, 2012). In considering many of these studies of masking and masking release, an underlying issue is whether the observed masking release is due to a reduction in energetic masking (EM) because the overlap of the stimuli in the auditory periphery is reduced by imposing the masking-release variable, or is due to a reduction in informational masking (IM; see review in Kidd *et al.*, 2008a) resulting from improved source segregation/selection or to an improvement in some other form of higher-level processing (e.g., better representation in memory or enhanced linguistic processing). Continuing with the example above about time-reversed masker speech, there are many facets of the masker speech that are altered by time reversal that could affect either EM or IM (e.g., changes in the envelope, an alteration in the normal statistical distribution of phoneme sequences, physiologically implausible production, etc., e.g., Rhebergen *et al.*, 2005). Again, similar arguments may be made about whether changes in EM or IM are responsible for the release from masking observed for other masking release variables such as spatial separation of sources or talker sex differences. The important point is that understanding the mechanisms responsible for release from SOS masking depends on determining how much EM is present in a given stimulus condition so that the subsequent effects of IM may be ascertained.

Although there are many differences in speech materials and procedures that may contribute to the disparate findings discussed above, it is possible nonetheless to identify two factors that appear to form the basis for many of these discrepancies. First, as has been widely appreciated and remarked upon dating even from the earliest work on the topic, speech masks other speech in multiple ways that involve different types and levels of processing by the listener. These include (as per Miller's quote above) the acoustic overlap of target and masker sources (and, by extension, overlap of the neural representations in the auditory system), the difficulty of perceptually segregating the sources and selectively attending to the target source (based on features that define the separate sources, *a priori* information about the sources, etc.), and the linguistic/semantic processing demands of the speech recognition task (which may apply to both target and masker sources to varying degrees; cf. Brouwer *et al.*, 2012). Even apparently minor differences in stimuli or procedures can affect which type of masking and processing demands influence performance. Second, as has been appreciated to a lesser degree and has not been remarked upon frequently in the literature, SOS masking experiments require a means for explicitly designating the target source distinct from competing sources. For example, if an observer in a listening experiment is presented with three concurrent voices how does the experimenter indicate which voice is the “target” to which the listener should attend and which voices are the “maskers” that should be ignored? Some means of target designation is necessary for the listener to understand how to solve the task (e.g., “pay attention to the talker on the right and not the talker on the left”; or, “attend to the male voice and ignore the female voice…”). The specific means of target source designation may interact with the other variables under study to affect the outcome of the experiment—particularly for the perceptual and linguistic variables noted above—and accordingly may strongly influence the interpretation of the results. The issue of target source designation typically is not relevant for the historically more common study of speech masked by noise where there is only one intelligible source and confusions or misdirected attention normally are not factors.

Because these two variables, the multiple levels of processing involved and the need for an explicit means of target source designation, may determine the outcome of the SOS masking experiment, comparisons across studies often are challenging. In many cases, it is unclear which cues are most effective in producing a release from SOS masking and whether the effectiveness of the cue is due to a reduction in EM or in IM. Furthermore, questions remain regarding the origins of the large intersubject differences found for SOS masking, both in terms of the amount of masking produced in the reference conditions (i.e., with limited source segregation cues) and how effectively individuals are able to exploit the different source separation cues. Is it possible for listeners to be categorized reliably according to these observed patterns of masking and masking release so that, for example, one subject shows a large masking release only when voice cues distinguish talkers while another subject shows a large benefit only when competing sources are spatially separated? Or, is it the case that listeners tend to differ in their ability to exploit *any* of the cues available to segregate sound sources?

The first goal of the current study was to measure the improvement in masked target speech identification produced by implementing three different masking release variables and, in each case, to determine the extent to which the consequent release from masking was due to a change in EM. The second goal was to determine whether consistent patterns of individual listener performance would be observed across the different masking-release conditions. The experimental approach chosen, including the target and masker speech and method of target speech designation, has been found to produce a high degree of IM in past studies thereby providing a large range over which to compare the efficacy of different masking release variables. Specifically, we used a closed-set matrix-style format similar to many others that have been used for both research and clinical purposes in the past (e.g., Spieth *et al.*, 1954; Hagerman, 1982; Wagener *et al.*, 1999; Bolia *et al.*, 2000). When used in SOS masking experiments, the target talker often is designated by a key word early in the sentence which the listener must recognize and associate with a specific talker. It is then assumed that the features distinguishing that voice are sufficient for the listener to track that talker's speech over time extracting the words uttered by the target talker while ignoring words spoken by competing talkers (cf. Brungart, 2001; Iyer *et al.*, 2010). When the target voice is similar to the competing voices and there are no other strong segregation cues, the ability to follow the target voice may not be reliable until the target is higher in level than the competing voices.

In the following experiments, a reference condition was tested in which, based on previous work, a high degree of IM was expected. Three stimulus variables were tested in conditions that were intended to provide a release from masking. The same group of listeners participated under each of these conditions so that the effectiveness of each masking release variable could be examined and comparisons made across the different conditions. These comparisons are of interest for determining individual differences among listeners and, because each of these variables could plausibly be used for the purpose of designating the target source among multiple sources, may reveal how different cues could lead to different experimental outcomes. Furthermore, a means for estimating the intelligibility of the target speech after accounting for the EM present under each condition was implemented using an ideal time-frequency segregation procedure (ITFS; Brungart *et al.*, 2006, 2009; see also “ideal binary mask” Wang, 2005; and related work by Anzalone *et al.*, 2006; Li and Louizou, 2007; Healey *et al.*, 2013). In this processing, the target and maskers were divided into spectro-temporal “tiles” and only the tiles in which the target energy exceeded the masker energy were retained. The assumption underlying this approach is that it tests the intelligibility of the target speech that remains after accounting for EM. This approach can be used to determine whether EM is altered by the masking-release variables and to estimate the amount of IM that is present under each stimulus presentation condition.

## II. METHODS

### A. Subjects

Six young adults (ages 21 to 30 years) participated in this experiment. Each subject had audiometric thresholds within normal limits for octave frequencies from 250 to 8000 Hz in both ears. The subjects received compensation for their participation in the experiments.

### B. Stimuli

The stimuli consisted of a set of 40 monosyllabic words divided into five syntactic categories: <name> <verb> <number> <adjective> <object> (Kidd *et al.*, 2008b). Within each category, there were eight exemplars. The words were spoken by 18 young-adult talkers with an equal number of males and females and the recordings were made by Sensimetrics Corporation (Malden, MA). All talkers recorded all words. The words were spoken individually with neutral inflection so that they could be concatenated in any order without the potential confound of across-word coarticulation effects. The corpus thus allowed for the construction of 32 768 (i.e., 8^5^) unique syntactically correct sentences per recorded talker. The sentences were used for both the “target” that was to be identified and the “maskers” that were to be ignored. The first word was used to designate the target talker and sentence (always the name “Sue”) while the remaining four words were chosen at random from the eight exemplars in each syntactic category and were scored. An 8 × 5 grid of the corpus of test words was displayed on the subject's monitor. The subjects were instructed to mouse-click the five words in order, one from each column left to right, comprising the target sentence. The words used for the sentences within a trial were always mutually exclusive as were the talkers.

### C. Procedures

The target sentence was always spoken by a female talker and presented from 0° azimuth (directly in front of the listener). There were either two or four masker sentences presented concurrently with the target sentence. In the reference condition (designated as the “baseline” condition), these masker sentences also were spoken by female talkers from 0° azimuth (i.e., same sex talkers, intelligible speech and colocated presentation). There were three other “comparison” conditions tested which were expected to produce a release from masking. These conditions were: (a) the use of male voices for the masker sentences (“male”), (b) the time-reversal of the words forming the masker sentences (“reversed”) on a word-by-word basis, and (c) the spatial separation of the masker sentences from the target sentence (“spatial”). In addition to the naturally produced speech, each of these four conditions was subjected to ITFS signal processing (e.g., Wang, 2005; Brungart *et al.*, 2006) that was used to exclude tiles (henceforth referred to as time-frequency [T-F] units) in which masker energy dominated target energy (i.e., where the target was presumably unavailable due to EM). The ITFS was intended to isolate the available target “glimpses” contributing to intelligibility after the masker-dominated T-F units were removed. After processing the remaining T-F units were reassembled and presented under the same four conditions as above to measure speech intelligibility. A more detailed description of this procedure is given in Sec. II D. This full set of conditions produced a total of 16 combinations [i.e., two signal processing conditions (natural or glimpsed) × two number-of-masker conditions (two or four) × four masker type conditions (baseline, male, reversed, spatial)].

During testing subjects were seated in individual double-walled sound-treated Industrial Acoustics Corporation (IAC) booths listening through Sennheiser HD280 Pro headphones (Sennheiser Electronic GmbH & Co. KG, Wedemark, Germany). For each condition a psychometric function was measured in which the target level was fixed at 55 dB sound pressure level and the masker level was varied to test six target-to-masker ratios (T/Ms) per condition. The sound levels were specified prior to the ITFS processing. The T/Ms were selected separately for each condition based on preliminary listening. Note that a T/M of 0 dB indicates that all talkers (target and individual maskers) were the same rms level prior to combination. Thus, the expected signal-to-noise ratio (SNR) would be specified as −3 dB for the 2-talker masker and −6 dB for the 4-talker masker.

The experimental session began with a block of the target sentences alone. This served the purpose of familiarizing the listeners with the sentence structure and response mode. All six subjects scored 100% correct on this first block of trials. The next step in the familiarization stage consisted of a written description of the experimental conditions with three example trials presented at descending T/Ms for each condition. No criterion was set for performance in these practice/example trials.

The experimental test blocks began immediately following the quiet testing and familiarization. Each 30-trial block that followed consisted of one of the 16 experimental conditions with a random ordering of five trials at each of the six T/Ms. The order of conditions tested in each 16-block set was randomized and four sets were completed. This resulted in 20 trials (80 scored words) at each T/M for each condition. Correct-answer feedback was given on all quiet, familiarization, and experimental trials. The entire experiment required 3–4 sessions of approximately 2 hours each.

### D. Signal processing/stimulus generation

The five-word target and masker sentences for all conditions were pregenerated and stored for playback during the experiment at a sampling rate of 44.1 kHz. The digital waveforms were D/A converted through an RME HDSP 9632 (ASIO) 24-bit sound card. All signal processing and experimental control was implemented via matlab (MathWorks Inc., Natick, MA).

In order to keep the processing the same in all conditions, the analysis and resynthesis required to produce the glimpsed conditions also was applied to the “natural” conditions. The ITFS analysis was the same as that used by Brungart *et al.* (2006, 2009) in which 128 frequency channels were analyzed with 20-ms windows (sequential windows overlapping by 10 ms). In our adaptation of that method, the frequency channels spanned 80 to 8000 Hz. In addition, all sentences were convolved with impulse responses that had been recorded in our laboratory via the Knowles Electronic Manikin for Acoustic Research (KEMAR) manikin for the source positions used in the experiment. Therefore, a version of the target and maskers was available separately for each ear.

All of the targets and the maskers, except for the maskers in the spatial condition, were processed for the source position of 0° azimuth. In the spatial condition, the masker placement was symmetric around the target. When there were two masker talkers the source positions were ±90° azimuth (to the left and right of the subject facing straight ahead) and when there were four masker talkers they were positioned at ±45° and ±90°. This step was also implemented prior to the ITFS analysis. Next, the masker sentences were summed for each ear and scaled to produce the desired T/M. The stimuli for each ear were then subjected to the ITFS analysis which resulted in a matrix of T-F values representing the energy in each of those units. For the natural speech conditions, all T-F units were retained in the resynthesis for both target and summed masker (an all ones binary mask; cf. Wang, 2005). For the glimpsed conditions, a local SNR criterion (LC; cf. Brungart *et al.*, 2006) value of 0 dB was applied such that the target energy was compared to the summed masker energy for each T-F unit and each ear separately. According to that algorithm, a 1 represents each unit containing target energy equal to or greater than the masker energy while a 0 is used for the remaining masker-dominated T-F units. Of the summed target and maskers for each ear, only the units with a value of 1 are retained while the units with a 0 are discarded. The synthesis is completed following application of the binary mask and the left ear and right ear signals are stored for playback. It is important to note that in the colocated conditions the targets and maskers all originated from 0° azimuth but still could have somewhat different resynthesized waveforms for the two ears due to small asymmetries in the KEMAR recordings.

**FIG. 1.**
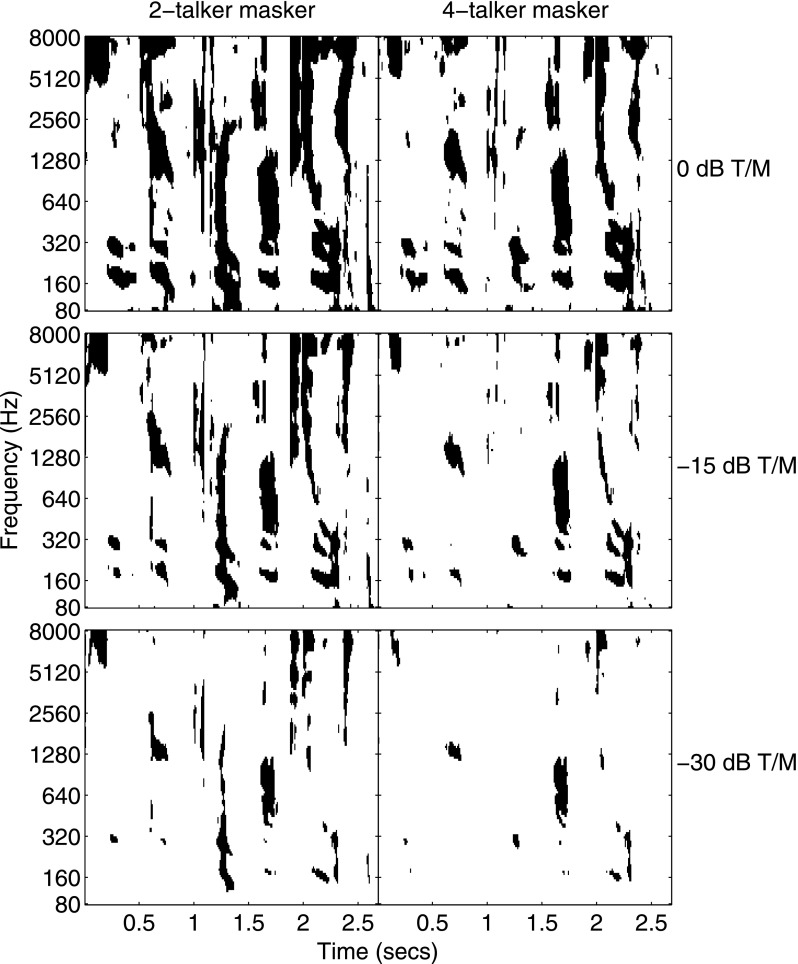
Schematic illustrations of the ITFS (see text) processing used in the baseline condition for 2-talker (left column) and 4-talker (right column) maskers. These plots illustrate the “binary masks” applied to the stimuli for target-to-masker ratios (T/Ms) of 0 dB (top row), −15 dB (middle row), and −30 dB (bottom row). The black regions indicate T-F units in which the target energy was equal to or greater than the masker energy and thus were retained in the processing while the white regions indicate T-F units that were dominated by masker energy (or were below the noise floor) and thus were removed by the processing.

Examples of the ITFS processing (for one ear) are shown in Fig. 1. The black regions represent values of 1 in the mask and therefore indicate the T-F units that were retained after processing. The left column shows the binary mask for the 2-talker masker baseline condition and the right column is for the 4-talker masker in the same condition. The rows are different T/Ms ranging from 0 to −30 dB. The target speech was the same in all panels. The masker speech was the same in each row but differed for the two columns in that the right column had two additional masker sentences. Note the difference in sparseness of the target-dominated speech with fewer T-F units remaining as T/M is decreased and, for each T/M, fewer T-F units for the 4-talker masker than for the 2-talker masker.

## III. RESULTS

**FIG. 2.**
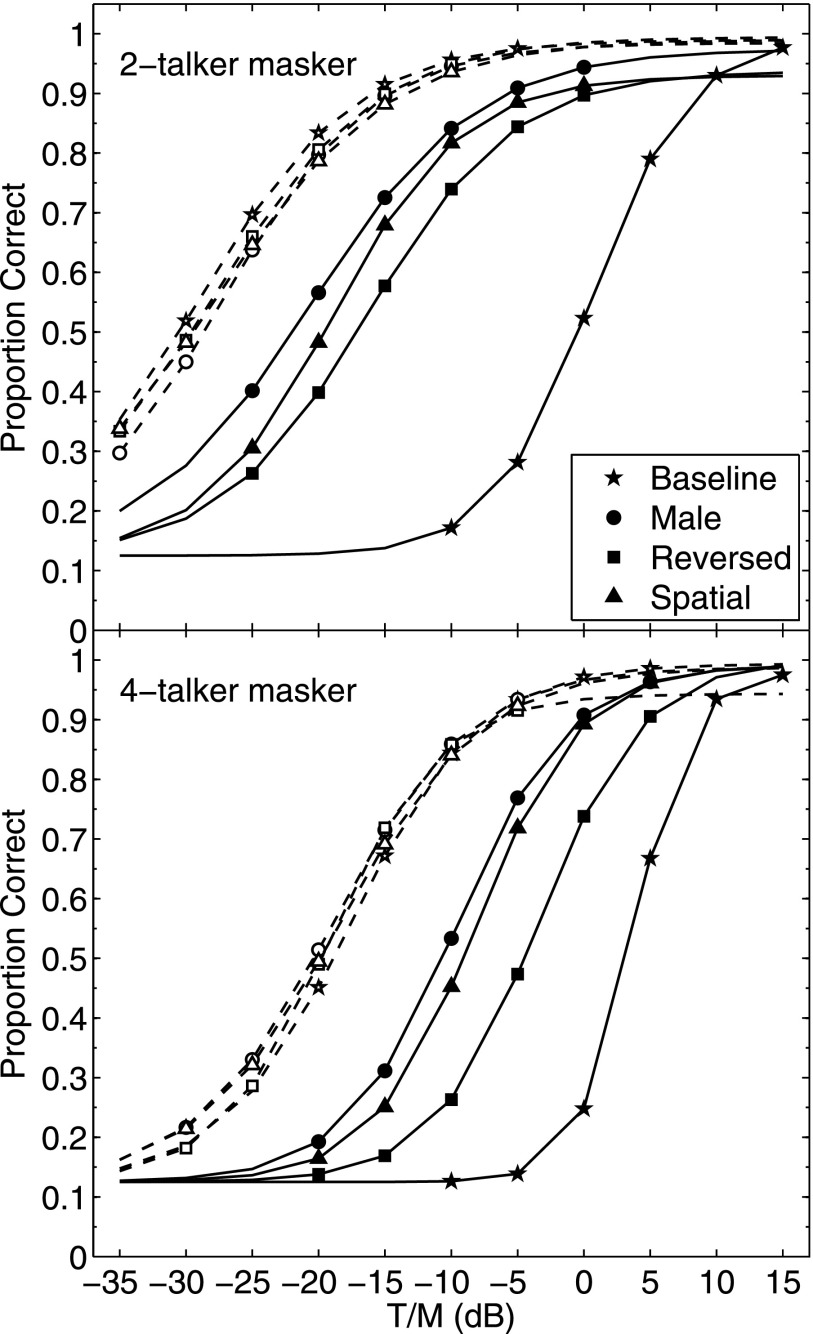
Performance-level functions for all conditions based on group mean values for the logistic fits to the individual listener data (see details in text). The upper panel contains the results from the 2-talker masker while the lower panel contains the results from the 4-talker masker. The symbols identify the different conditions and are placed only at the T/M values that were tested, with the dashed lines showing performance for the glimpsed stimulus conditions and the solid lines showing performance for the natural speech conditions.

The results yielded performance-level functions for each listener and condition. The data were individually fit with logistic functions so as to obtain an estimate of the threshold, defined as the T/M in dB for 50% correct word identification. The thresholds for individual listeners and the group means are given in Table I. In order to obtain group average performance-level functions, the means of the parameters of the individual fits were used to construct logistic functions for each condition. The group mean performance-level functions are shown in Fig. 2 both for the 2-talker masker (upper panel) and for the 4-talker masker (lower panel). Within each panel, the solid lines and values for the filled symbols were computed for the baseline and three masking release conditions for natural speech while the dashed lines with open symbols are the four average functions for the glimpsed speech. The symbols used to identify the different conditions are placed only at the values of T/M that were tested, which varied by condition. It is clear from this figure that performance varied dramatically across conditions with the baseline condition displaced the furthest to the right along the abscissa, the three comparison conditions falling along intermediate values of T/M, and all of the glimpsed conditions tightly clustered at the leftmost (negative) T/M values. This order of conditions is apparent for both the 2- and 4-talker masker cases although all 4-talker masker psychometric functions are shifted to the right, by varying amounts, relative to their 2-talker masker counterparts. The slopes of the fitted functions were somewhat steeper for the 4-talker masker conditions than for the 2-talker masker conditions and within each masker the natural baseline condition was steepest with the other three natural conditions and all glimpsed conditions having similar slopes. The average slopes for the 2-talker masker ranged from 3.2%/dB to 4.2%/dB in all cases except for the natural baseline condition which had a slope of 6.3%/dB. For the 4-talker masker the range was 4.0%/dB to 6.1%/dB with a slope of 10.5%/dB for the natural baseline condition. These steeper slopes for the natural baseline conditions likely reflect the contributions of a level cue when the target became the dominant (highest level) source.

**FIG. 3.**
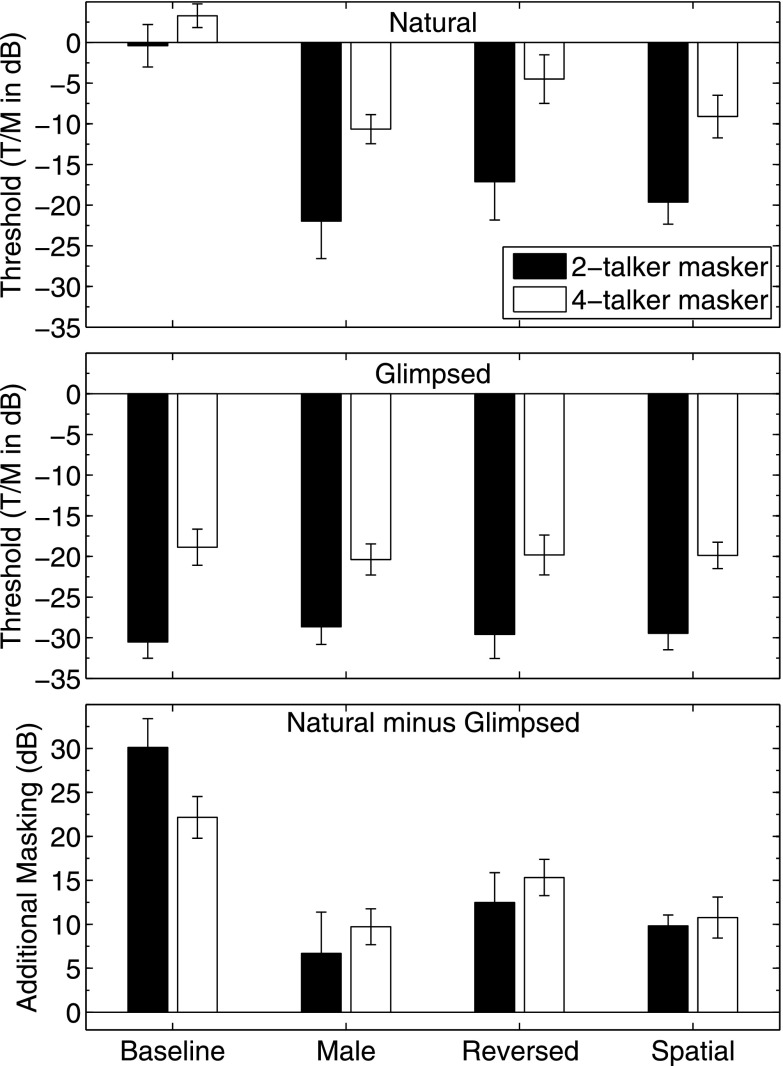
Group mean T/Ms at threshold in decibels and standard errors of the means computed from the performance-level functions for all conditions tested for the natural (upper panel) and glimpsed (middle panel) 2- and 4-talker maskers (black and white bars, respectively). The differences between the natural and glimpsed thresholds (“additional masking”) are shown in the lower panel.

The trends described above for performance in each condition may be summarized by comparing the thresholds. The group mean T/Ms at threshold and standard errors of the means extracted from the individual logistic fits are plotted in Fig. 3. The upper panel contains the data for the natural speech stimuli and the middle panel contains the results from the glimpsed speech stimuli. The lower panel is the difference between the thresholds obtained for the natural speech stimuli shown in the upper panel and the corresponding glimpsed speech stimuli shown in the middle panel. These values are referred to as “additional masking” and will be discussed later. The masker conditions are shown in pairs of bars along the abscissa (left to right): baseline, male, reversed, and spatial, while the ordinate (upper and middle panels) is the T/M at threshold in dB. Each pair of bars contains the data for the 2- (black) and 4-talker (white) masker cases.

First, with respect to the T/Ms at threshold for the natural speech 2-talker masker, the group mean value for the baseline condition was about −0.4 dB. One subject, L2, achieved a threshold of −5.5 dB while the remaining subjects fell within a narrow range from −0.7 to 1.4 dB (Table I). The corresponding group mean thresholds for the comparison conditions were much lower than for the baseline condition for the 2-talker masker. They were −21.8, −17.1, and −19.6 dB, respectively, for the male, reversed, and spatial conditions. These thresholds yield release-from-masking amounts of 21.4, 16.7, and 19.2 dB, respectively, as referenced to the baseline threshold.

For the 4-talker masker, the group mean threshold for the baseline condition was 3.3 dB. The comparison conditions produced average thresholds of −10.7, −4.5, and −9.1 dB for the male, reversed, and spatial conditions with corresponding reductions in masking of 14, 7.8, and 12.4 dB, respectively. The thresholds obtained for the 4-talker maskers were higher in all cases than the corresponding thresholds measured under the 2-talker maskers. However, the increase in threshold differed across conditions. For baseline, the threshold in the 4-talker masker was only about 3.7 dB higher than for the 2-talker masker whereas the thresholds for male, reversed, and spatial maskers were 11.1, 12.6, and 10.5 dB higher for the 4-talker masker than for the 2-talker masker, respectively. This differential increase, which affected the baseline reference condition less than the comparison conditions, meant that the reduction in masking for the three comparison conditions relative to baseline was less in all cases for the 4-talker masker. Because T/M is based on the level per talker, the overall level of the 4-talker masker was 3 dB greater than for the 2-talker masker (3 dB higher in SNR). This increase in overall masker level could account for the increase for the baseline condition of 3.7 dB noted above but does not explain the much greater increases for the three comparison conditions. However, it should be noted that the point at which the target talker becomes the highest-level source (0 dB T/M) or higher than the sum of the masker talkers (0 dB SNR which is 3 dB T/M for two masker talkers and 6 dB T/M for four masker talkers) may effectively limit the magnitude of the observed masking release. This point is considered in more detail in Sec. IV.

For the glimpsed speech 2-talker maskers, the effect of masker type was negligible. The threshold T/M was −30.6 dB in the baseline condition, −28.7 dB for the male condition, −29.6 dB for the reversed condition, and −29.4 dB for the spatial condition. These thresholds for the glimpsed speech were lower in all cases than the corresponding thresholds for the natural speech. The differences between the glimpsed baseline and comparison conditions were much smaller—less than 2 dB—than were found for the natural speech conditions and the glimpsed baseline had the lowest threshold of the group. Thus, there were no instances of “release” from masking within the glimpsed conditions. The minor effect of masker type for the glimpsed 4-talker masker condition was similar to that noted for the 2-talker maskers with average thresholds of −18.7, −20.4, −19.8, and −19.9 dB T/M for baseline, male, reversed, and spatial conditions, respectively. The three comparison conditions were thus slightly lower than baseline but all four thresholds fell within a narrow range of about 1.7 dB. The increases in threshold observed for the glimpsed 4-talker masker conditions compared to the glimpsed 2-talker masker conditions were 11.9, 8.3, 9.8, and 9.5 dB for the baseline, male, reversed, and spatial masker conditions, respectively. When compared to the increases found for the natural speech conditions the largest difference was for the baseline condition while the other comparison conditions increased by roughly the same amount.

An analysis of variance with repeated measures on the thresholds contained in Table I revealed significant main effects of speech type (natural vs glimpsed) [F(1,5) = 265.2; p < 0.001], number of maskers (two vs four) [F(1,5) = 298.1; p < 0.001] and masker condition (baseline, male, reversed, and spatial) [F(3,15) = 154.1; p < 0.001]. These effects are apparent in Fig. 3. The significant 2-way interaction of speech type by masker condition [F(3,15) = 6.2; p = 0.006] is also clearly evident in that figure. The interaction of number of maskers by masker condition [F(3,15) = 180.3; p < 0.001] likely reflects the result that the effect of adding two masker talkers is different for the baseline masker condition. It is also clear in Fig. 3 that speech type and number of maskers do not interact [F(3,15) = 0.21; p = 0.67]. The three-way interaction, however, was significant [F(3,15) = 17.9; p < 0.001] and again could be due to the baseline condition being different in regards to two versus four masker talkers, but only for Natural speech and not for Glimpsed speech. In order to obtain a better understanding of these findings each of the four subdivisions of the experiment (2- vs 4-talker maskers, natural vs glimpsed) were analyzed further by conducting separate analyses in which the single within-subjects factor of masker type was evaluated. The results indicated that for both of the natural speech conditions, masker type was significant: For the 2-talker masker, F(3,15) = 148.35; p < 0.001 and for the 4-talker masker, F(3,15) = 125.54; p < 0.001. For the two glimpsed speech conditions, masker type was *not* significant: with F(3,15) = 1.64; p = 0.223 for the 2-talker masker and F(3,15) = 2.22; p = 0.13 for the 4-talker masker.

The lack of significance of masker type for the glimpsed conditions, while a negative statistical result, is important because it is consistent with the interpretation that EM (i.e., as defined by masker energy > target energy within a T-F unit; note that this definition does not include interactions among adjacent T-F units or binaural interaction within units^1^) was the same across conditions. It is of interest, then, to consider each natural masker condition with respect to the *additional* masking—predominantly IM (cf. Brungart *et al.*, 2006)—it created. The lower panel of Fig. 3 plots the results of these calculations. For both 2-talker and 4-talker maskers, the greatest additional masking was observed in the baseline condition, which was about 30 dB for the two talker masker and about 20 dB for the 4-talker masker. The ordering of the comparison conditions with respect to this additional IM was the same for both two and four maskers (from most to least): reversed, spatial, and male. The greater IM found for the reversed condition is of particular interest because it has the least linguistic similarity of the three comparison conditions and, given that it was not intelligible speech, would not be expected to produce a high proportion of masker confusions. This point is considered further in Sec. IV.

**FIG. 4.**
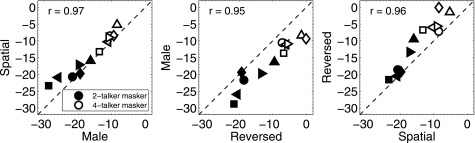
Thresholds from individual subjects (coded by symbols) plotted for each pair of natural speech comparison conditions. The three panels show the individual data plotted for the following pairs of conditions: male vs spatial (left); reversed vs male (center); spatial vs reversed (right). The data from both the 2- and 4-talker maskers are plotted in each panel with filled and open symbols, respectively.

With respect to the performance of individual subjects, there were some notable trends in the data. Figure 4 shows individual thresholds for the natural speech target and maskers for the three comparison conditions plotted each against the other. These comparisons are of interest because they indicate the extent to which listeners were successful in using different source segregation cues to reduce the high IM present in the baseline condition. It was possible, for example, that some listeners would rely more heavily on one strategy or cue than another or that, in contrast, “good listeners” could effectively use any cue that is available and “poor listeners” could not. A hallmark of IM is large individual differences (cf. Kidd *et al.*, 2008a), but the extent to which subjects tend to exploit one particular cue or strategy in preference to others is not well-established. Figure 4 illustrates the range of individual differences in thresholds for this subject group for both numbers of maskers. Thresholds for all subjects are higher for four maskers than for two maskers and the data points generally are distributed along the diagonal indicating a tendency for individual subjects to be characterized according to their ability to use any segregation cue to overcome IM. The correlations based on all 12 data points (six subjects by two numbers of maskers) were 0.97, 0.95, and 0.96 for male vs spatial, reversed vs male, and spatial vs reversed, respectively. Although the number of subjects is too small to draw any broad conclusions about subject categories, the data are more in line with subject performance that is consistently good or bad across the different cues rather than subjects obtaining a strong advantage from one of the cues while not receiving a large advantage from a different cue.

### A. Error analysis

**FIG. 5.**
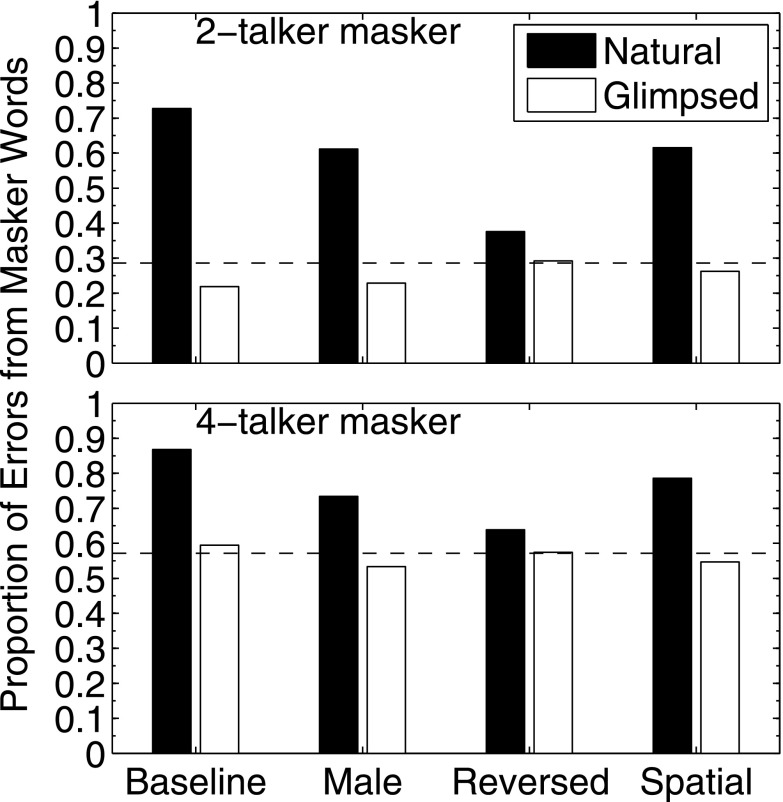
Proportions of the total errors that were masker errors tabulated for the 2-talker masker (upper panel) and the 4-talker masker (lower panel) averaged across the six T/M values used in each condition. The four masker conditions (baseline, male, reversed, and spatial) are indicated along the abscissa. For each type of masker, the filled bars show natural speech conditions while the open bars show glimpsed speech conditions. The horizontal dashed line in each panel indicates the proportion of masker errors expected by chance.

The patterns of mistakes made by listeners engaged in the speech identification task can be used to draw inferences about the underlying mechanisms of masking. Confusions between target words and masker words are associated with high IM while randomly distributed errors are indicative of EM-dominated conditions (cf. Brungart, 2001; Kidd *et al.,* 2005; Brungart *et al.*, 2006; Iyer *et al.*, 2010). The proportions of masker confusion errors occurring in the speech identification task computed across all subjects and T/Ms in all 16 conditions tested here are shown in Fig. 5. The 2-talker masker condition is shown in the upper panel and the 4-talker masker is shown in the lower panel.

The four masker conditions are indicated along the abscissa and the ordinate is the proportion of all responses that matched one of the masker alternatives presented for that word position/category. Both natural (black/filled bars) and glimpsed (white/open bars) are shown. The dashed horizontal line in each panel is the proportion of errors that would be expected to occur from guessing. Note that chance is different for the 2-talker masker (2/7 for expected masker errors) than for the 4-talker masker (4/7).

The assumption underlying analysis of these error patterns is that conditions dominated by EM would result in a proportion of masker errors governed simply by chance—which would correspond to values on or near the horizontal dashed lines. For the glimpsed speech conditions, the T-F units dominated by masker energy are removed leading to the hypothesis that the errors that occurred would be due to insufficient information to identify the target (EM) but not enough information about the masker to yield confusions (IM). Thus, performance after processing reflects EM because the target energy that is “covered up” by the masker is unavailable to the listener and so the errors should be randomly distributed. Inspection of Fig. 5 supports this interpretation. In all of the glimpsed conditions the error proportions fall very near the chance performance value (horizontal dashed line). In contrast, for the natural speech maskers, the errors are dominated more by masker confusions for the baseline, spatial, and male conditions. For the reversed condition, the errors are much closer to the chance line as would be expected from unintelligible speech. However, it is striking that, despite the near-chance error patterns, the thresholds for the reversed condition were higher than for either the male or spatial conditions (cf. Fig. 3) where the error patterns were dominated by confusions. As discussed in more detail in Sec. IV, the presence of significant “additional masking” for the reversed condition together with near-chance masker confusions indicates that explicit confusions with masker items are not necessary to cause substantial IM; the additional masking for the reversed condition was more than 15 dB on average for the 2-talker masker condition (Fig. 3, lower panel).

## IV. DISCUSSION

The current study demonstrated that three cues fostering the perceptual segregation of a target stream of speech from competing masker streams of speech produced large reductions in masking. Thus, a sex difference between target and masker talkers, masker time reversal and spatial separation of sources all significantly reduced the masking found in the baseline condition. These reductions in masking relative to the baseline condition were as much as 20 dB for the 2-talker masker and 14 dB for the 4-talker masker, on average, for this group of listeners. Furthermore, based on the results of the glimpsing analysis, we conclude that the differences in performance across the natural (unprocessed) speech conditions were not due to differences in EM produced by the different masker manipulations. This conclusion is supported by the finding that the thresholds for all four glimpsed conditions for either two or four masker talkers were not significantly different and fell within narrow ranges. Those thresholds were about 30 dB lower than the natural speech baseline threshold for the 2-talker masker and about 20 dB lower for the 4-talker masker. It should be emphasized that the magnitude of the release from masking observed under the various conditions depends crucially on the amount of masking that is present in the reference condition to begin with; here, that was the baseline condition where the target talker was identified by association of a key word with a specific voice early in the target sentence. Following the target stream of speech after the keyword (the scored test items) required the listener to track the target talker by voice or, potentially at the higher T/Ms, by loudness/saliency.

The implication of these findings for the issue of target source designation raised in Sec. I is that employing one of these source segregation cues to designate the target (e.g., instructing the subject to: “…report the words spoken by the female talker in the group of male talkers…” or “…report the words from the talker that is straight ahead and ignore the talkers to the sides…”) may greatly reduce the IM that is present and therefore lessen the effect of any additional cue in releasing masking. Although only three such cues were tested in the current study, other variables could exert a similar influence on a SOS masking experiment. Determining and studying how a “target” source is designated in natural conversation outside of the laboratory can be a very complex problem and may depend on many variables, and their interactions, that are difficult to control and to quantify. For example, the target source typically varies with turn taking in conversation and new sources may join or leave a conversation unexpectedly, creating a significant degree of uncertainty in communication. The *a priori* and ongoing information available to a listener (i.e., context) may have a significant impact on the amount of IM that is present as well as how effectively the listener can exploit the various cues available (e.g., visual information) to solve the task.

**FIG. 6.**
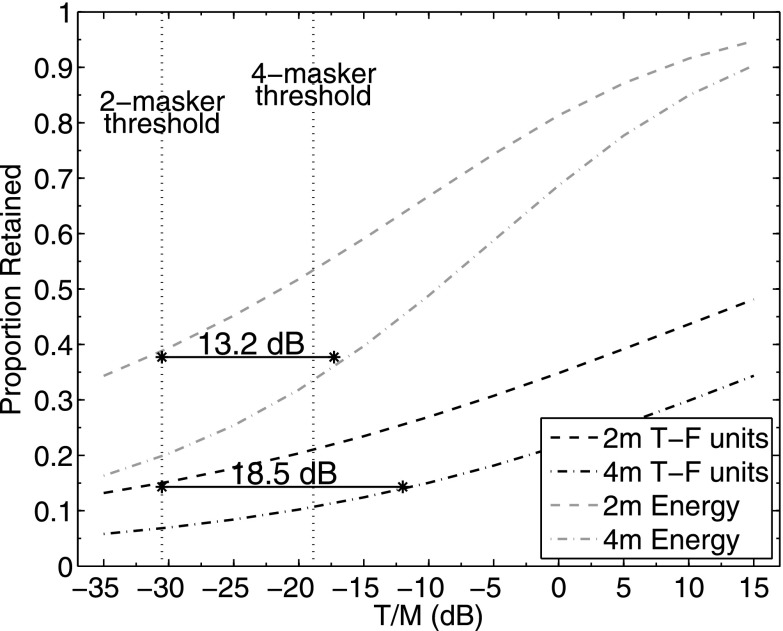
Proportion of the Glimpsed target retained as a function of T/M averaged across conditions for 2- and 4-talker maskers. The light lines (upper two functions) are values for the proportion of target energy retained while the heavy lines (lower two functions) show the proportion of T-F units retained. The dashed lines are for the Glimpsed target in the 2-talker masker and the dash-dot lines are for the 4-talker maskers. The pair of vertical dotted lines intersect the abscissa at the threshold T/M for the 2-talker masker (left) and the 4-talker masker (right). The solid horizontal lines and corresponding decibel values indicate the difference in dB between the proportion of energy (13.2 dB; upper line) or T-F units (18.5 dB; lower line) retained at threshold for the 2-talker masker and the same proportion for the 4-talker masker (see text).

The magnitude of the reduction in masking (relative to baseline) caused by the three segregation cues tested here was greater when the masker comprised two talkers—where it averaged about 19 dB across the three comparison conditions—than when it comprised four talkers where the average reduction in masking dropped to about 11 dB. To the extent that these large masking releases reflect a release from IM, the findings are consistent with a variety of other studies that show that IM generally is greatest for a small number of competing sources when EM is relatively low. For example, a similar conclusion has been reached for the effect of IM found in multi-tone masking conditions where the maximum IM occurs for relatively few components (e.g., Neff and Green, 1987; Oh and Lutfi, 1998; review in Kidd *et al.*, 2008a). In all cases tested here, the amount of masking increased as the number of independent masker talkers increased from two to four. Similar findings with respect to the number of competing masker talkers have been reported by Brungart *et al.* (2001) and Brungart *et al.* (2009). Other than the baseline conditions, the increase in threshold due to the increase in the number of masker talkers exceeded the 3 dB higher SNR for the 4-talker masker. Some insight into the additional masking caused by the 4-talker masker may be found by considering the information that remains in the glimpsed stimulus as estimated here through ITFS processing. At each T/M, the glimpsed stimuli contain only the T-F units that are likely to contribute significantly to identification performance (depending on the LC value chosen; cf. Brungart *et al.*, 2006). Thus, there is a direct relationship between the proportion of T-F units retained after ITFS processing and speech identification performance. We verified this direct and orderly relationship for the glimpsed stimuli used in the present study. The additional increase in masking (above the 3 dB increase in SNR) for the 4-talker masker relative to the 2-talker masker may be explained by the higher proportion of T-F units that are energetically masked. The 4-talker masker generally obscures more T-F units than the 2-talker masker at an equivalent T/M (cf. Fig. 1). For example, for the glimpsed 2-talker masker conditions, the proportion of target-dominated T-F units at threshold (averaged across the four masker conditions; T/M = −30.5 dB) was about 0.15 while the proportion of energy retained by those points was about 0.38. For the same proportion of points to be retained for the 4-talker masker, the T/M must be increased by 18.5 dB while achieving the same proportion of energy retained requires an increase in T/M of 13.2 dB. The apparent discrepancy between the two values is due to the property of the ITFS analysis that retains the higher-level points at any given T/M. So the high-level points present at the lowest T/M are preserved as the target level increases causing the slope of the function relating the proportion of energy retained in the glimpsed stimulus to differ from the corresponding function relating proportion of T-F units retained over the range of values of interest here. These functions and the computations just described are illustrated in Fig. 6. In this figure, the abscissa is T/M in dB and the ordinate is proportion of T-F units (heavy lines) or energy (light lines) retained for both 2- and 4-talker maskers. If we assume that the thresholds measured in our observers for the glimpsed stimulus are related to a constant proportion of target energy that remains after ITFS processing (0.38 for the 2-talker masker as shown in Fig. 6), then the increase in T/M necessary to provide that same proportion of energy retained for the 4-talker masker (13.2 dB) is roughly comparable to the increase in thresholds that was measured in the glimpsed-speech experiment which ranged from 9.5 to 11.9 dB across masker conditions. Because this increase is related to EM, by definition (T-F units where T > M), this suggests that the difference in thresholds between 2- and 4-talker maskers is primarily a consequence of increased EM. The reduced magnitude of the release from masking for the 4-talker masker relative to the 2-talker masker conditions may be due to this increase in EM or, perhaps more specifically, to an increase in the proportion of EM to IM. It is possible too that the reduced masking release for the 4-talker masker simply reflects a compression of the range of thresholds bounded by EM at the lower T/Ms and the use of a level segregation cue at the upper end where the target level exceeds the masker level in the baseline condition. This phenomenon of range compression in SOS masking is another factor that must be considered when comparing results across studies (cf. related discussion in Best *et al.*, 2013).

Applying ITFS processing, as in the current study, eliminates T-F units according to a specified T/M (or SNR) criterion within each unit. As discussed by Brungart *et al.* (2006, 2009), this process affects both EM and IM. The T-F units dominated by masker energy are, by definition, “energetically masked.” However, there is an important theoretical—and practical—issue concerning the contributions made by individual T-F units to overall performance depending on whether IM also is present. Theories attempting to predict speech intelligibility in noise (e.g., Articulation Index theory; cf. Egan and Wiener, 1946; French and Steinberg, 1947; Kryter, 1962) make the assumption that each frequency band within the relevant time frame^2^ contributes to the overall intelligibility depending on the SNR and other factors such as a weighting or importance function across frequency. If the SNR is very low the channel makes a small (often set to zero) contribution to the overall score. So, removing a channel contributing zero to the AI would have no practical effect. That practice is well-suited for the most commonly considered case of speech masked by Gaussian noise. However, for SOS masking conditions high in IM, the presence of a masker-dominated T-F unit may actually *decrease* overall intelligibility well beyond any additional EM it may cause by interacting with adjacent units (cf. Freyman *et al.*, 1999; Brungart *et al.*, 2006). Consider the difference in thresholds between the glimpsed and natural masker conditions. Adding in the T-F units dominated by the maskers (e.g., Fig. 3, lower panel) has a profoundly different effect depending on the IM value of the masker. Some increase in thresholds would be expected by adding in the masker-dominated T-F units due to spread of EM to target-dominated T-F units (for that reason, the values in the lower panel of Fig. 3 are referred to as “additional masking” rather than IM). Using a (high EM) noise masker control, Brungart *et al.* (2006) found about a 3–5 dB increase in thresholds when the T-F units excluded with an LC of 0 dB (as here) were present in the stimulus. The additional masking found in their study for speech maskers—more than 10 dB in some cases—was attributed to IM. In this study we also have the case where adding T-F units dominated by the masker not only does not add to the overall intelligibility, but, depending on the IM value of the masker-dominated T-F units, decreases intelligibility considerably.

Viewed from the perspective of the increase in masking due to the addition of the T-F units dominated by the masker, the reversed masker had the greatest IM value of any condition except baseline. It should be pointed out, though, that the additional masking (natural speech relative to glimpsed) for the spatial condition may be underestimated somewhat because of the possibility that binaural interaction (an EM release) contributed to the spatial release from masking (natural speech spatial condition relative to baseline). That case is unique among those tested here because the interaural differences between target and maskers could contribute some release from EM from binaural analysis.^1^ Although there are clear linguistic effects in SOS masking (e.g., Freyman *et al.*, 2001, 2004; Van Engen and Bradlow, 2007; Brouwer *et al.*, 2012; Kidd *et al.*, 2014) it is interesting that the unintelligible reversed speech masker was so effective *without* producing explicit confusions. Here, as much as 15 dB of additional masking was produced on average for the reversed masker (cf. 4-talker masker, lower panel Fig. 3). Indeed, time-reversed maskers have been used as (relatively) high IM/low EM maskers in some studies in the past (Best *et al.*, 2007). It is possible that reversed speech is more difficult to segregate than different sex talkers or spatially separated sources because it cannot be excluded (“filtered”) according to any simple perceptual property and, in fact, retains many of the important properties of natural speech (e.g., Kellogg, 1939; Cherry, 1953). In contrast, the male masker could be excluded based on the perception of one or more simple acoustic differences (e.g., fundamental frequency, formant properties, etc.) related to the sound sources (e.g., de Cheveigne *et al.*, 1995; Hafter and Saberi, 2001; Kidd *et al.*, 2003; Carr-Levy, 2010). Similarly, differences in the apparent locations of the sound sources—physical differences in azimuth causing interaural disparities—could be used to filter out the masker (e.g., Teder-Salejarvi and Hillyard, 1998; Arbogast and Kidd, 2000; Marrone *et al.*, 2008).

There is evidence suggesting that the reversed masking release does operate at a relatively high level of processing. Newman *et al.* (2015), for example, have reported a significant reversed masking release in children as young as 4 years old although newborn infants do not appear to exhibit differential responses to time-forward vs reversed speech (Newman, 2009). Thus, there is a developmental component likely related to language acquisition that underlies this difference. So, rather than excluding time-reversed speech according to a distinction along a single perceptual dimension related to a simple acoustic property, it may be that reversed speech is rejected only at higher levels of linguistic processing. It is interesting to note that a similar experienced-based development of linguistically based IM has been reported by Ezzatian *et al.* (2010) for masking caused by speech in a non-native language.

It seems likely that the IM caused by the reversed condition would be akin to that found for masker speech presented in a language not understood by the listener (e.g., Ezzatian *et al.*, 2010; Iyer *et al.*, 2010; Brouwer *et al.*, 2012; Calandruccio *et al.*, 2013). In both cases many of the source-specific acoustic features are retained while meaning is eliminated (see Freyman *et al.*, 2001, for similar observations). The similarity of the EM produced by all three conditions here, as reflected in the glimpsed thresholds, argues against any significant difference between conditions other than the IM they create. It would be interesting to determine whether speech in languages not understood by the listener would also produce similar EM (cf. Rhebergen *et al.*, 2005, for related discussion and counter example). Our findings of substantial additional masking from reversed speech lead to the question of whether the IM for the reversed condition could be further reduced if it were combined with an additional segregation cue. Although we did not test that hypothesis in the current study, Swaminathan *et al.* (2015) reported results that are relevant to this question. In a study of the benefit of musical training on SOS masking, they reported a reversed masking release of about 15 dB using methods similar to those used in this study that was further reduced almost 5 dB by ±15° spatial separation of two masker talkers from the target talker.

## V. SUMMARY AND CONCLUSIONS

The first conclusion from the current findings is that the underlying amount of EM was nearly identical (total range of 1.9 dB for 2-talker maskers and 1.7 dB for 4-talker maskers) in all natural speech conditions: the baseline reference and the three comparison conditions causing release from masking. This conclusion is based on the similar performance of the group of listeners on the ITFS-processed speech across all masking conditions over a range of T/Ms. Furthermore, an analysis of the proportion of energy retained in the glimpsed T-F units showed a rough correspondence to the difference in thresholds between the 2- and 4-talker maskers that could be attributed to differences in EM. Thus, the ITFS processing approach provided a means for quantifying differences in EM across experimental conditions, suggesting that this type of analysis may be useful in other applications where it is desirable to separate EM from IM.

The second conclusion is that each of the masking release variables: different sex talkers, spatial separation of sources and time-reversal, produced a large release from IM. One important implication of this finding is that, while any of the three variables could be used to designate the target source separate from the masking sources in a SOS experiment, doing so would have a significant effect on the extent to which further masking release could be observed with other cues.

The third conclusion is that the amount of masking beyond the performance obtained on the ITFS-processed speech (additional masking, largely IM) varied considerably across conditions. For the comparison conditions, the largest amount of additional masking (and correspondingly the least amount of release) was for the time-reversed masker (however, see caveat regarding potential release from EM in the spatial condition noted above).^1^ Thus, large amounts of IM are possible even in the absence of explicit confusions among target and masker words and for speech maskers that are not intelligible.

Finally, large individual differences were found for all natural speech conditions. Although the subject group was too small to support firm conclusions, the subjects who showed a large masking release for one variable were likely to show large masking release for the other two variables as well, consistent with the notion that listeners fall along a continuum of a general ability to use source segregation cues to release IM (cf. Neff and Dethlefs, 1995).

## Figures and Tables

**TABLE I.** Individual and group mean thresholds (T/M in dB) for all conditions (see text).

Listener	Glimpsed				Natural			
	Baseline	Male	Reversed	Spatial	Baseline	Male	Reversed	Spatial
2-talker masker								
1	−32.1	−29.8	−33.6	−29.9	0.7	−20.8	−18.6	−20.7
2	−30.3	−28.2	−30.4	−31.0	−5.5	−28.8	−21.6	−23.4
3	−27.5	−29.4	−27.1	−26.0	1.0	−16.3	−9.5	−15.9
4	−32.5	−29.0	−28.3	−29.4	1.4	−19.4	−19.2	−19.8
5	−32.4	−30.9	−32.3	−31.6	−0.7	−25.9	−20.5	−20.9
6	−28.9	−24.6	−26.0	−28.4	0.5	−19.7	−13.4	−17.1
Mean	−**30.6**	−**28.7**	−**29.6**	−**29.4**	−**0.4**	−**21.8**	−**17.1**	−**19.6**
Standard error	**0.85**	**0.89**	**1.22**	**0.82**	**1.06**	**1.89**	**1.92**	**1.11**
4-talker masker								
1	−21.0	−22.4	−24.2	−20.9	2.6	−10.8	−7.0	−8.3
2	−19.1	−19.8	−19.2	−20.0	1.0	−13.7	−6.7	−13.2
3	−16.8	−17.3	−17.6	−18.2	3.4	−8.4	−1.4	−5.2
4	−18.2	−19.8	−17.9	−19.2	5.4	−9.4	−0.1	−8.4
5	−20.7	−22.3	−20.8	−22.6	3.9	−11.1	−6.0	−10.3
6	−16.4	−20.7	−19.3	−18.6	3.4	−10.9	−5.6	−9.0
Mean	−**18.7**	−**20.4**	−**19.8**	−**19.9**	**3.3**	−**10.7**	−**4.5**	−**9.1**
Standard error	**0.78**	**0.78**	**0.99**	**0.67**	**0.59**	**0.73**	**1.20**	**1.08**

## Footnotes

^1^ The release from masking found for the natural speech spatial condition relative to baseline likely involved a small amount of binaural analysis causing a reduction in EM. One estimate of the magnitude of this effect may be found in Marrone *et al.* (2008) who reported a spatial release from masking of about 1.5 dB (group average) for two independent speech-shaped noise maskers (typically used as a high-EM control) separated from a target talker by ±90°. A slightly larger spatial release (3.6 dB) was reported by Kidd *et al.* (2010) for noise maskers separated from a diotic speech target by interaural time differences of ±600 *μ*s. Note that the interaural differences in the target-dominated T-F units are retained in the glimpsed spatial condition but are not retained in the masker-dominated T-F units which were removed in the ITFS processing. Thus, the glimpsed spatial condition does not reflect any improvement in T/M that might be gained from binaural analysis in the masker-dominated T-F units.

^2^ AI computations are typically based on long-term averages within frequency channels. We have not attempted to compute AI-like quantities for the T-F units considered in this study. However, the principle of independently contributing frequency channels considered over brief time intervals (i.e., “dip-listening” in masker envelope minima) has been established for various stimuli and conditions (cf. Rhebergen *et al.*, 2006; Wan *et al*., 2014).
